# Use of an HRP2-based rapid diagnostic test to guide treatment of children admitted to hospital in a malaria-endemic area of north-east Tanzania

**DOI:** 10.1111/j.1365-3156.2011.02737.x

**Published:** 2011-02-14

**Authors:** George Mtove, Behzad Nadjm, Ben Amos, Ilse C E Hendriksen, Florida Muro, Hugh Reyburn

**Affiliations:** 1 National Institute for Medical Research, Amani CentreMuheza, Tanzania; 2 London School of Hygiene and Tropical MedicineLondon, UK; 3 Teule HospitalMuheza, Tanga, Tanzania; 4 Mahidol-Oxford Research UnitBangkok, Thailand; 5 Kilimanjaro Christian Medical CentreMoshi, Tanzania

**Keywords:** malaria, severe, rapid diagnostic test, hospital

## Abstract

**Objective:**

To compare the performance of the Paracheck™ rapid diagnostic test (RDT) with microscopy for diagnosing malaria in hospitalised children.

**Methods:**

Children aged between 2 months and 13 years with fever were enrolled in the study over 1 year. A standard clinical history and examination were recorded and blood drawn for culture, complete blood count, Paracheck™ RDT and double-read blood slide.

**Results:**

Of 3639 children enrolled, 2195 (60.3%) were slide positive. The sensitivity and specificity of Paracheck were 97.5% (95% CI 96.9–98.0) and 65.3% (95% CI 63.8–66.9), respectively. There was an inverse relationship between age-specific prevalence of parasitaemia and Paracheck specificity. In logistic regression model, false-positive Paracheck results were significantly associated with pre-admission use of antimalarial drug (OR 1.44, 95% CI 1.16–1.78), absence of current fever (OR 0.64, 95% CI 0.52–0.79) and non-typhi *Salmonella* bacteraemia (OR 3.89. 95% CI 2.27–6.63). In spite of high sensitivity, 56/2195 (2.6%) of true infections were Paracheck negative and 8/56 (14.3%) were in patients with >50 000 parasites/μl.

**Conclusions:**

Paracheck had poor specificity in diagnosing malaria in severely ill children; this was likely to be due to HRP2 persistence following recent parasite clearance. The combination of positive Paracheck and negative blood slide results identified a group of children at high risk of non-typhi *Salmonella* infection. While Paracheck was highly sensitive, some high-density infections were missed. For children with severe febrile illness, at least two reliable negative parasitological test results should be available to justify withholding antimalarial treatment; the optimal choice of these has yet to be identified.

## Introduction

The use of rapid immunochromatographic tests for malaria (RDTs) in primary care facilities has been studied over a number of years, and these tests are now being rolled out on a large scale in Africa as a means to restrict antimalarial drug use to parasitologically confirmed cases, a policy now supported by WHO (WHO 2010). However, the WHO criteria for malaria diagnosis apply irrespective of severity, and it is therefore surprising that as far as we are aware, only one small study has so far been published on the use of RDTs in patients admitted to hospital (Birku *et al.* 1999).

The wider use of RDTs in patients hospitalised with severe febrile illness seems inevitable given the increasing availability of RDTs. Given current evidence of the low accuracy of routinely read slide results, this may represent an improvement over current practice (Reyburn *et al.* 2004; Zurovac *et al.* 2006). However, the performance of HRP2-based RDTs may differ when used for severely ill children compared to use in non-severe illness. First, specificity may be reduced by the persistence of HRP2 for up to 5 weeks following clearance of parasites and this may be a particular concern in patients who are frequently infected or who have recently taken antimalarial drugs, both of which may be more likely in hospitalised children in malaria-endemic areas (Wongsrichanalai *et al.* 1999; Swarthout *et al.* 2007). Secondly, it has been suggested that test sensitivity may paradoxically decline at very high parasite densities (more common in severe than non-severe malaria) because of flooding of RDT capture sites (Reyburn *et al.* 2007). Thirdly, antibodies to HRP2 that are acquired with increasing exposure to malaria might result in age-dependent test performance (Biswas *et al.* 2005). And finally, the interpretation of a combination of a positive RDT result and negative blood slide may indicate ‘recent malaria’ and this has been associated with certain bacterial infections in severely ill children that may provide added diagnostic value if both RDT and blood slide results are available (Brent *et al.* 2006; Nadjm *et al.* 2010).

In this study, we compared results of a commonly used HRP2-based RDT (Paracheck™) with those from double-read research slides in guiding the care of children enrolled in a 1-year study of children admitted to a district hospital for febrile illness in an area of intense malaria transmission. We compare the technical performance of Paracheck with research-quality blood slide results and suggest how HRP-2-based RDT results might contribute to clinical care in African district hospitals.

## Methods

### Study site and data collection

The study was conducted in a district hospital in north-eastern Tanzania serving a predominantly rural population with childhood mortality that is typical for Tanzania (165 deaths/1000 person years under the age of 5 years). The area is highly endemic for *Plasmodium falciparum* (*P. falciparum*) malaria.

Details of the study have been published elsewhere (Nadjm *et al.* 2010). Briefly, over the course of 1 year, all daytime paediatric admissions were screened for inclusion and were eligible if aged 2 months to 13 years with axillary temperature ≥37.5 °C or a history of fever within the previous 48 h. Children with chronic illness except HIV or admitted with trauma or a surgical condition were excluded.

After consenting procedures, a standard clinical history and examination based on IMCI guidelines were recorded by a study clinician (WHO 2000a). Pulse oximetry was used on a finger or toe and height and weight were measured. Lumbar puncture was undertaken on suspicion of meningitis according to WHO criteria. Venous blood was drawn for point of care (POC) tests of haemoglobin concentration, blood glucose (Hemocue™, Anglholm, Sweden), blood lactate (Lactate-Pro™; Arkray Inc, Kyoto, Japan), HRP-2-based RDT for *P. falciparum* (Paracheck™; Orchid Biomedical, Mumbai, India) and HIV antibody tests (Capillus HIV-1, HIV-2 Test; Trinity Biotech, Ireland and Determine HIV-1/2 Test; Abbott Laboratories, IL, USA). Blood was sent to the laboratory for full blood count (Act/Dif™; Beckman-Coulter) and aerobic blood culture (BactAlert™; Biomerieux, France) with identification of organisms by standard means. Blood slides were stained with Giemsa and independently double-read with discordant results resolved by a third reader. Paracheck tests were stored in a ventilated room out of direct sunlight with temperature documented not to exceed 40 °C as recommended by the manufacturer.

### Data management and analysis

Data were scanned using Teleforms (Verity software Inc.) into MS-Access (Microsoft Corp, Redmond, VA, USA) and analysed using Stata-10 (Stata Corp, College Rd, TX, USA). Final blood slide results are considered as the ‘reference standard’ result in making comparisons with Paracheck results throughout the paper.

Statistical tests used chi-squared for comparison of proportions and *t*-test (for parametric variable) or rank-sum (for non-parametric variable) for comparison of normally or non-normally distributed data, respectively. The logistic model was constructed with ‘positive RDT and negative blood slide’ as the dependent variable and factors in Table 3 as independent variables. All of the initially chosen independent variables were retained in the final model.

**Table 3:** Pathogenic bacteria isolated from blood or CSF by category of Paracheck and blood slide results

	NTS	Other organism	Total
Slide negative and RDT negative	41 (4%)	102 (11%)	943
Slide negative and RDT positive	67 (13%)	31 (6%)	501
Slide positive, P.f. density <5000/μl	23 (6%)	10 (2%)	405
Slide positive, P.f. density 5000–50 000	13 (1%)	18 (2%)	917
Slide positive, P.f. density 50 000+	16 (2%)	20 (2%)	873
Total	160 (4%)	181 (5%)	3639

NTS, non-typhi *Salmonella*; RDT, rapid diagnostic test.

### Ethics

The study was approved by the Ethics Committees of the National Institute for Medical Research, Tanzania (NIMR/HQ/R.8a/Vol.IX/392), and the London School of Hygiene and Tropical Medicine, UK (LSHTM Ethics # 2087). Witten informed consent to participate was obtained from the parent or guardian of each child in the study.

## Results

**Figure 1:**
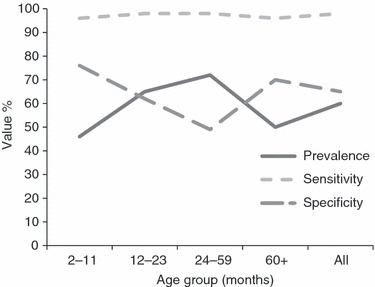
Age-specific sensitivity and specificity of rapid diagnostic test results by prevalence of a positive blood slide of children in the study.

After exclusions for missing data, 3639 children were included in the analysis, all of whom had a blood slide and Paracheck result. Overall, 2139 (58.8%) were both *P. falciparum* slide positive and Paracheck positive, and 943 (25.9%) were negative to both tests. Of the 557 discordant results, 501/557 (90%) were Paracheck positive but slide negative and 56/557 (10%) were Paracheck negative and slide positive. Using the blood slide result as the reference standard, there was an inverse relationship between age-specific prevalence of parasitaemia and the specificity of Paracheck (Table 1, Figure 1).

**Table 1:** Age-specific sensitivity, specificity and predictive values of Paracheck compared to blood slide results in 3639 children in the study

	Prevalence of slide positive	Sensitivity % (95% CI)	Specificity % (95% CI)	Positive predictive value % (95% CI)	Negative predictive value % (95% CI)
2–11 months	487/1054 (46.2%)	96.4 (95.3–97.5)	75.5 (72.3–78.1)	77.3 (74.8–79.8)	96.0 (94.9–97.2)
12–23 months	740/1139 (65.0%)	97.7 (96.8–98.6)	62.2 (59.3–65.0)	82.7 (80.5–84.9)	93.6 (92.2–95.0)
24–59 months	811/1134 (71.5%)	98.2 (97.4–99.0)	48.9 (46.0–51.8)	82.8 (80.6–85.0)	91.3 (89.7–93.0)
60+ months	157/312 (50.3%)	96.2 (94.1–98.3)	70.3 (65.3–75.4)	76.7 (72.0–81.3)	94.8 (92.3–97.3)
Total	2195/3639 (60.3%)	97.5 (96.9–98.0)	65.3 (63.8–66.9)	81.0 (79.8–82.3)	94.4 (93.7–95.1)

The sensitivity of Paracheck was above 95% in detecting *P. falciparum* infections with >2000 parasites/μl with no consistent trend with increasing density above this level. The sensitivity of Paracheck was lower in detecting infections with <2000 parasites/μl and especially infections with <200 parasites/μl compared to infections with >2000/μl (Table 2). However, low-density infections were relatively uncommon; only 20/2139 (0.9%) and 240/2, 139 (11.2%) of positive blood slide results were at densities of <200 and <2000 parasites/μl, respectively. More than half of all false-negative Paracheck results (30/56, 53.6%) were in children with a parasite density of >2000 parasites/μl and 8/56 (14.3%) were in children with parasite densities greater than 50 000/μl.

**Table 2:** Sensitivity of Paracheck results by parasite density of reference blood slide results

Parasite density/μl	Prevalence	RDT positive	RDT negative	Sensitivity (95% CI)
1–199	20/1464 (1.4%)	16	4	80.0 (78.0–82.1)
200–1999	220/1664 (13.2%)	198	22	90.0 (88.6–91.4)
2000–4999	165/1609 (10.3%)	159	6	96.4 (95.5–97.3)
5000–49 999	917/2361 (38.8%)	901	16	98.3 (97.7–98.8)
50 000–200 000	693/2137 (32.4%)	689	4	99.4 (99.1–99.7)
>200 000	180/1624 (11.1%)	176	4	97.8 (97.1–98.5)

RDT, rapid diagnostic test.

An invasive bacterial infection was isolated in 341/3639 (9.4%) children in the study and these were more common in slide-negative (241/1444, 16.7%) compared to slide-positive children (100/2195, 4.6%, *P* < 0.001). Similarly, bacterial infection was more common in RDT-negatives than in RDT-positives; 194/2640 (7.4%) of RDT-positive and 147/999 (14.7%) of RDT-negative children had invasive bacterial disease (*P* < 0.001). However, of the 501 children who were slide negative but Paracheck positive, 98 (19.6%) had invasive bacterial disease and 67 (69.1%) of these were caused by non-typhi *Salmonella* (NTS) infections (Table 3).

Overall, 597/1850 (32.3%) of children with a true-positive Paracheck result were reported to have taken an antimalarial drug in the 2 days prior to admission compared to 188/443 (42.4%) of children with a false-positive Paracheck result (*P* < 0.001). Factors judged likely to be associated with false-positive Paracheck results were assessed in the logistic regression model in Table 4.

**Table 4:** Factors associated with the combination of a negative blood slide and positive Paracheck result

	*N* (prevalence, %)	Unadjusted OR (95% CI)	*P*	Adjusted OR (95% CI)	*P*
Age 12–23 months	1073 (29.5)	1.00 (0.78–1.28)	0.96	1.06 (0.81–1.39)	0.430
Age 24–59 months	2254 (61.9)	1.12 (0.90–1.42)	0.36	1.22 (0.93–1.59)	0.160
Age 60+ months	312 (8.6)	1.14 (0.79–1.63)	0.48	1.35 (0.92–2.00)	0.130
Days ill, OR per day		1.10 (1.01–1.04)	0.002	1.01 (1.00–1.03)	0.113
Antimalarial in last 2 days	1102 (34.8)	1.46 (1.19–1.80)	<0.001	1.44 (1.16–1.78)	0.001
Non-typhi *Salmonella*	169 (4.4)	5.05 (3.63–7.03)	<0.001	3.82 (2.25–6.50)	<0.001
Any invasive bacterial disease	341 (9.4)	2.90 (2.24–3.74)	<0.001	1.52 (0.99–2.33)	0.054
Fever >37.5 °C	2383 (65.6)	0.69 (0.57–0.84)	<0.001	0.64 (0.52–0.79)	<0.001

## Discussion

The main finding of the study was the low specificity of Paracheck compared to reference blood slides; the overall figure was among the lowest recorded for Paracheck and in children aged between 2 and 5 years, specificity dropped below 50%. The proportion of Paracheck results that were false-positives correlated closely with age-specific parasite prevalence and recent antimalarial drug use but was not independently associated with age.

The rate of false-positive Paracheck results is at least partly a function of the time between parasite clearance and disappearance of HRP2 from blood and this can last for more than 5 weeks (Swarthout *et al.* 2007) and is likely to be associated with recent antimalarial treatment. One would expect this to be particularly common in children who are admitted to a district hospital because this constitutes the first referral level of care, and in our study, we found that over one-third of all children in the study and almost half of the children with a false-positive Paracheck were reported to have taken an antimalarial drug in the 2 days prior to admission. In addition, apparent low specificity may be the result of Paracheck or other RDTs exceeding blood slide results in sensitivity; studies by both Bell *et al.* (2005) and Hopkins *et al.* (2008) have found that a substantial proportion of results that were negative to expert slide reading but positive to Paracheck were positive when tested by polymerase chain reaction (PCR). Without recourse to PCR, we are unable to replicate this result but it seems likely that at least some of the apparent low specificity of Paracheck that we observed was the result of Paracheck detecting submicroscopic parasitaemia.

The influence of antibodies to HRP2 on the accuracy of RDTs is not clear. On the assumption that HRP antibodies accelerate the disappearance of reactive HRP2, one would expect RDT sensitivity and specificity to increase with increasing exposure, for which age is a reasonable proxy in a stable population such as that in our study area. This is supported by the findings of (Fryauff *et al.* 1997) who found a marked difference in sensitivity over and under the age of 10 years among residents of a malaria-endemic area of Irian Jaya, although in our study population, with a much narrower age range, we did not observe age-specific trends in sensitivity. Biswas *et al.* (2005) studied HRP2 levels and antibody titres to HRP2 in 45 subjects in a low-transmission area for up to 6 weeks following infection with *P. falciparum* and found that HRP2 antigen remained elevated for at least 7 days post-treatment, despite the development of HRP2-specific immune responses. Our findings were thus consistent with the conclusions of Biswas *et al.*, that antibody levels to HRP2 are unlikely to exert an important effect on test results in children with severe febrile illness.

The relatively low specificity of Paracheck in our study suggests that its use in a hospital setting will result in significant overuse of antimalarial drugs with the possible neglect of alternative diagnoses. Brent *et al.* (2006) previously described the strong association between a false-positive RDT result and blood stream bacterial infections caused by NTS and other Gram-negative organisms and thus the combination of a negative blood slide result and positive Paracheck result should alert clinicians to the possibility of these infections. Given the currently unsatisfactory clinical predictors of bacterial infection in children admitted to hospital in resource-poor settings, this could be a useful diagnostic aid, and a positive HRP2 RDT result should not deter clinicians from providing presumptive treatment with antimicrobials, especially if the blood slide is negative.

By contrast, Paracheck reached very high levels of sensitivity and negative predictive values approached 100%, a finding consistent with other studies (Hopkins *et al.* 2007; Laurent *et al.* 2010). As expected from results of the recent WHO-sponsored evaluation of RDTs, sensitivity dropped below 90% for the detection of low-density infections but otherwise was consistently above the minimum level of recommended level of 95% (WHO 2000a, 2008). The relative importance of low-density infections varies by their prevalence, and low-density infections are more common in low-transmission areas and in asymptomatic individuals; in a community survey in a low-transmission area of the Solomon Islands, Harris *et al.* (2010) found that almost half of all *P. falciparum* infections were with <100 parasites/μl. This is in contrast to hospitalised children in our study where <1% of infections were in this category. Thus, in spite of the high sensitivity of Paracheck in detecting infections above 2000 parasites/μl, more than half of the false-negative Paracheck results in our study were in children with >2000 parasites/μl and almost one in six of the false-negative results was in children with high-density (>50 000 parasites/μl) infections. False-negative RDT results at high density have been described by at least two other studies although the explanation is still not clear (Reyburn *et al.* 2007; Laurent *et al.* 2010). The lack of association with increasing parasite density in our study suggests that flooding of RDT capture sites with excess antigen is an unlikely explanation. Other explanations include mutations in the HRP locus similar to those that have been found in South America (Gamboa *et al.* 2010).

In practice, the small number of false-negative RDT results that we observed suggests that it would be unwise to withhold antimalarial treatment on the basis of a single negative Paracheck result in a severely ill child. Ideally, at least one other parasitological test result should be used and results should ideally be available before starting treatment. This could be a second RDT, preferably based on the detection of alternative antigen to HRP2 such as lactate dehydrogenase (LDH) or a rapidly read quality-controlled blood slide; while the latter is clearly preferable, the limitations on laboratory quality in Africa create serious challenges. More research is needed on what is sufficient evidence to justify withholding antimalarial treatment in a severely ill child admitted to hospital in malaria-endemic areas.

In conclusion, HRP-2 devices in hospitalised children in high-transmission settings suffer from low specificity that is largely dependent on the risk of recently cleared *P. falciparum* infection. Thus, in areas of intense transmission of *P. falciparum*, patients may be overtreated with antimalarial drugs and a positive HRP2 test result should not discourage presumptive treatment with antimicrobial drugs. The combination of a positive HRP2 test with a negative blood slide result may suggest an increased risk of invasive Gram-negative septicaemia. While the Paracheck results in this study reached high levels of sensitivity, a small number of high-density *P. falciparum* infections were recorded as Paracheck-negative, suggesting that at least one more quality-controlled parasitological test should be used before withholding antimalarial treatment in patients with suspected severe malaria. The choice of test or combination of tests to guide treatment of children admitted to hospital with suspected malaria has not so far been defined.
